# Tracking copper nanofiller evolution in polysiloxane during processing into SiOC ceramic

**DOI:** 10.1107/S1600576724003133

**Published:** 2024-06-18

**Authors:** Patricia A. Loughney, Paul Cuillier, Timothy L. Pruyn, Vicky Doan-Nguyen

**Affiliations:** a Materials Science and Engineering The Ohio State University Columbus OH43212 USA; b Materials and Manufacturing Directorate Air Force Research Laboratory Wright-Patterson Air Force Base OH45433 USA; HPSTAR and Harbin Institute of Technology, People’s Republic of China

**Keywords:** polymer-derived ceramics, polysiloxane, X-ray total scattering, pair distribution function, nanofillers

## Abstract

Polymer-derived ceramics (PDCs) remain at the forefront of research for a variety of applications including ultra-high-temperature ceramics, energy storage and functional coatings. Despite their wide use, questions remain about the complex structural transition from polymer to ceramic and how local structure influences the final microstructure and resulting properties. This is further complicated when nanofillers are introduced to tailor structural and functional properties, as nanoparticle surfaces can interact with the matrix and influence the resulting structure. The inclusion of crystalline nanofiller produces a mixed crystalline–amorphous composite, which poses characterization challenges. With this study, we aim to address these challenges with a local-scale structural study that probes changes in a polysiloxane matrix with incorporated copper nanofiller. Composites were processed at three unique temperatures to capture mixing, pyrolysis and initial crystallization stages for the pre-ceramic polymer. We observed the evolution of the nanofiller with electron microscopy and applied synchrotron X-ray diffraction with differential pair distribution function (d-PDF) analysis to monitor changes in the matrix’s local structure and interactions with the nanofiller. The application of the d-PDF to PDC materials is novel and informs future studies to understand interfacial interactions between nanofiller and matrix throughout PDC processing.

## Abstract 2

The structural changes occurring during pyrolysis from pre-ceramic polymer to polymer-derived ceramic are traditionally challenging to characterize, and the inclusion of nanoparticle filler throughout the matrix complicates this further. In this work, the authors demonstrate the value of synchrotron X-ray scattering and pair distribution function analysis to track these structural changes with isolation of nanoparticle–matrix interactions at the local scale.

## 1. Introduction

In the past ten years, the number of review and perspective articles on polymer-derived ceramics (PDCs) has increased dramatically compared with previous decades (Ackley *et al.*, 2023 ▸; Loughney *et al.*, 2022 ▸; Sujith *et al.*, 2021 ▸; Ren *et al.*, 2021 ▸; Xia *et al.*, 2020 ▸; Barrios & Zhai, 2020 ▸; Lu, 2015 ▸; Mera *et al.*, 2015 ▸; Bernardo *et al.*, 2014 ▸). This is due to their inherent advantages over conventional ceramics, including enhanced processability into unique form factors and potential to improve control and tunability on the nanostructure scale. Tunable structure lends itself to tunable properties, making PDCs interesting candidates for several applications including ultra-high-temperature ceramics (Ionescu *et al.*, 2019 ▸), functional coatings (Lu & Erb, 2018 ▸; Barroso *et al.*, 2019 ▸; Tavares *et al.*, 2014 ▸), biomedical devices (Arango-Ospina *et al.*, 2020 ▸), battery systems (Knozowski *et al.*, 2021 ▸; Mukherjee *et al.*, 2019 ▸; Loughney *et al.*, 2022 ▸) and catalyst supports (Loughney & Doan-Nguyen, 2020 ▸; Araldi Silva *et al.*, 2021 ▸; Schumacher *et al.*, 2020 ▸) with the inclusion of functional nanofillers. Variables that allow this structural tunability include polymer composition (backbone, sidechain), processing conditions (tem­perature, heating rate, environment) and the use of additives (transition metal centers, nanofillers) (Colombo *et al.*, 2010 ▸; Bernardo *et al.*, 2009 ▸). Each variable influences the final composition and structure, concentration of free carbon, and resulting materials properties. Thus, discernment of structure–processing–property relations remains at the forefront of research. However, despite the uptick in fundamental research, several questions remain concerning (1) the complex structural transitions occurring during the polymer to ceramic transition on a local scale and (2) characterization methods that probe both crystalline and amorphous components when interfacing PDCs with nanofillers for advanced nanocomposites.

A pre-ceramic polymer (PCP) is subject to dramatic physical changes during processing into a PDC. This begins with crosslinking and gas evolution at temperatures removing most of the organic component (Sorarù *et al.*, 2002 ▸). The result is dramatic volume shrinkage and densification as the tem­perature increases during the transition, often leading to void and crack formation (Colombo *et al.*, 2010 ▸). Structurally, the changes a PCP undergoes are also dramatic. After crosslinking and outgassing, pyrolysis occurs. For polysiloxanes that become SiOC PDCs, pyrolysis transforms the material from a crosslinked polymer into an amorphous ceramic, structurally understood as a covalent network of silicon tetrahedra bonded randomly to oxygen and carbon, made up of SiC_4_, SiC_3_O, SiC_2_O_2_, SiCO_3_ and SiO_4_ (Lu & Erb, 2018 ▸). This is commonly probed using solid-state nuclear magnetic resonance (Hung *et al.*, 2021 ▸). As the temperature increases, a transition from amorphous to crystalline ceramic occurs, whose onset temperature depends on several variables such as precursor composition, filler composition and processing conditions. During this transition, it is understood that networks of graphitic carbon begin to form alongside phase-separated regions of amorphous SiO_2_ (Lu & Erb, 2018 ▸). As the temperature increases, SiO_2_, SiC and graphitic carbon domains further separate and crystallize, forming a final composition often described as SiO_2(1−*x*)_C_*x*_ + *y*C_free_ (Lu & Erb, 2018 ▸; Sorarù *et al.*, 2002 ▸). Crystallization is observed by the emergence of Bragg peaks in X-ray diffraction (XRD) patterns (Wang *et al.*, 2015 ▸; Lu & Erb, 2018 ▸; Lu, 2015 ▸; Bernardo *et al.*, 2014 ▸). The temperature range during which this occurs depends on the amount and species of free carbon formed during pyrolysis (Sorarù *et al.*, 2002 ▸). Characterization of carbon species is possible through a combination of Raman spectroscopy and X-ray photoelectron spectroscopy, as demonstrated by Wang *et al.* (2015 ▸). Carbon evolution is heavily influenced by starting precursors such as PCP backbone structure and functional groups (Key *et al.*, 2022 ▸), transition metal additives as filler (Yang & Lu, 2021*b* ▸), and processing conditions such as ramp rate, temperature and gas flow (Barrios & Zhai, 2020 ▸).

The addition of nanofiller into the PDC matrix is a viable option to both disperse functional properties in the PDC and impede detrimental crack and void formation. Filler allows for pathways for organic gas to escape without the need to form voids and adds an unshrinkable component to the matrix to help with densification and volume shrinkage (Colombo *et al.*, 2010 ▸). However, the inclusion of additives complicates the resolution of structural changes occurring during transformation from amorphous to crystalline ceramic, as the additive can react and evolve with increased temperatures. Efforts to understand the effect of metal additives on the structural changes to the PDC during processing have included low-temperature processing to induce crosslinking (Martin *et al.*, 2021 ▸) and an investigation of the influence of additives on the formation of different phases after processing to the ceramic (Yang & Lu, 2021*b* ▸). In both cases, these metal additives are metal centers introduced in the early stages with the polymers. Not as well understood is the structural evolution occurring at the interface between metal nanoparticle additives and the matrix, as the formation of such an interface creates opportunities for surface reactions that influence the resulting structure and tunable properties. XRD has often been used to track the formation of intermetallic species at elevated temperatures. However, an intermetallic shell of considerable size is necessary to meet the Bragg conditions and thus be discernible from a laboratory XRD source (10–50 nm). New nucleated phases and the diffusion pathways necessary are much smaller and occur much earlier than when laboratory XRD visibility is achieved. As a result, information is lost on the kinetics of these transformations, and even the existence of metastable phases if growth and diffusion stop before a considerably sized shell is formed. To achieve a complete understanding of the nucleated phases, resulting structure and tunable properties and overall control of the system, characterization methods that simultaneously probe average and local structure changes in an amorphous matrix dispersed with crystalline nanoparticles should be applied.

X-ray total scattering is a promising characterization technique for PDCs containing a combination of amorphous and crystalline components. The crystalline nanofillers contribute sharp Bragg peaks to the diffraction pattern, while the locally ordered amorphous PDCs and liquid PCPs contribute broad diffuse scattering features (Takeshi & Billinge, 2003 ▸). Total scattering considers both Bragg and diffuse components. Often, a Fourier transform is applied to interpret the reciprocal-space scattering signal as a real-space pair distribution function (PDF) with peaks corresponding to frequent inter­atomic distances (Proffen *et al.*, 2003 ▸). The shortest inter­atomic distances give information about bonding environ­­ments and local ordering inaccessible by traditional XRD, while larger interatomic distances give information about the crystalline components with long-range order. Application of PDF analysis to PDCs shows potential to elucidate the mechanisms by which the polymer to amorphous and crystalline ceramic transitions occur on the local scale. These transitions in particular have been cited as unclear in several reviews, and resolution of this issue is a listed topic in several ‘future work’ sections of papers (Schiţco *et al.*, 2016 ▸; Yang & Lu, 2020 ▸; Loughney *et al.*, 2022 ▸; Ionescu *et al.*, 2019 ▸). As far as we are aware, there are only a few examples of PDF analysis being applied to PDCs (Yang *et al.*, 2021 ▸; Heinemann *et al.*, 1999 ▸; Schiţco *et al.*, 2016 ▸). Schiţco *et al.* (2016 ▸) compare PDF signatures from three unique PCPs (polysiloxane, polysilazane and polycarbosilane) that have gone through pyrolysis up to 750°C in two different gas environments. Bonds probed include Si—O from α-SiO_2_, Si—C from β-SiC and Si—N from α-Si_3_N_4_, as well as tetrahedral bonds from SiO_4−*x*_C*_x_*, SiC_4_, SiN_4_ and SiN_4−*x*_C*_x_*. Yang *et al.* (2021 ▸) use electron diffraction to extract radial distribution functions of the remaining amorphous components for 1400°C-processed SiOC samples. However, with electron microscopes and laboratory X-ray diffractometers, the accessible momentum transfer (*Q* = 2π/*d*) is typically insufficient to obtain PDFs with real-space resolution capable of differentiating between Si—O, Si—C and C—C bond distances. Synchrotron PDF analysis provides the necessary resolution for probing the bonding environments in mixed amorphous–crystalline PDCs with nanofillers. In addition, the high signal-to-noise ratio enables earlier detection of crystallization, as demonstrated in a recent synchrotron XRD study of polysiloxane processed up to 1200 and 1500°C (Lu & Chaney, 2023 ▸). Crystallized SiO_2_ was found at 1200°C, before the crystallization of SiC, which challenges the previously established understanding of the phase separation mechanism during PCP pyrolysis into PDC (Lu & Chaney, 2023 ▸). PDF analysis shows promise to monitor the evolution of the interface between added nanofiller and the polysiloxane matrix with a data processing technique known as differential PDF (d-PDF) (Kofalt *et al.*, 1986 ▸; Petkov *et al.*, 2002 ▸; Terban & Billinge, 2022 ▸). In one example, d-PDF analysis was used to detect solvent restructuring around a nanoparticle core by subtracting the neat solvent signal from the mixed solvent–nanoparticle signal (Zobel *et al.*, 2015 ▸). The residual signal contained contributions from only the nanoparticles and their influence on the solvent. This technique has never been applied to a PDC with nanofiller system and will provide insight into the interactions between the nanofiller and the matrix throughout the processing steps.

**Figure 1:**
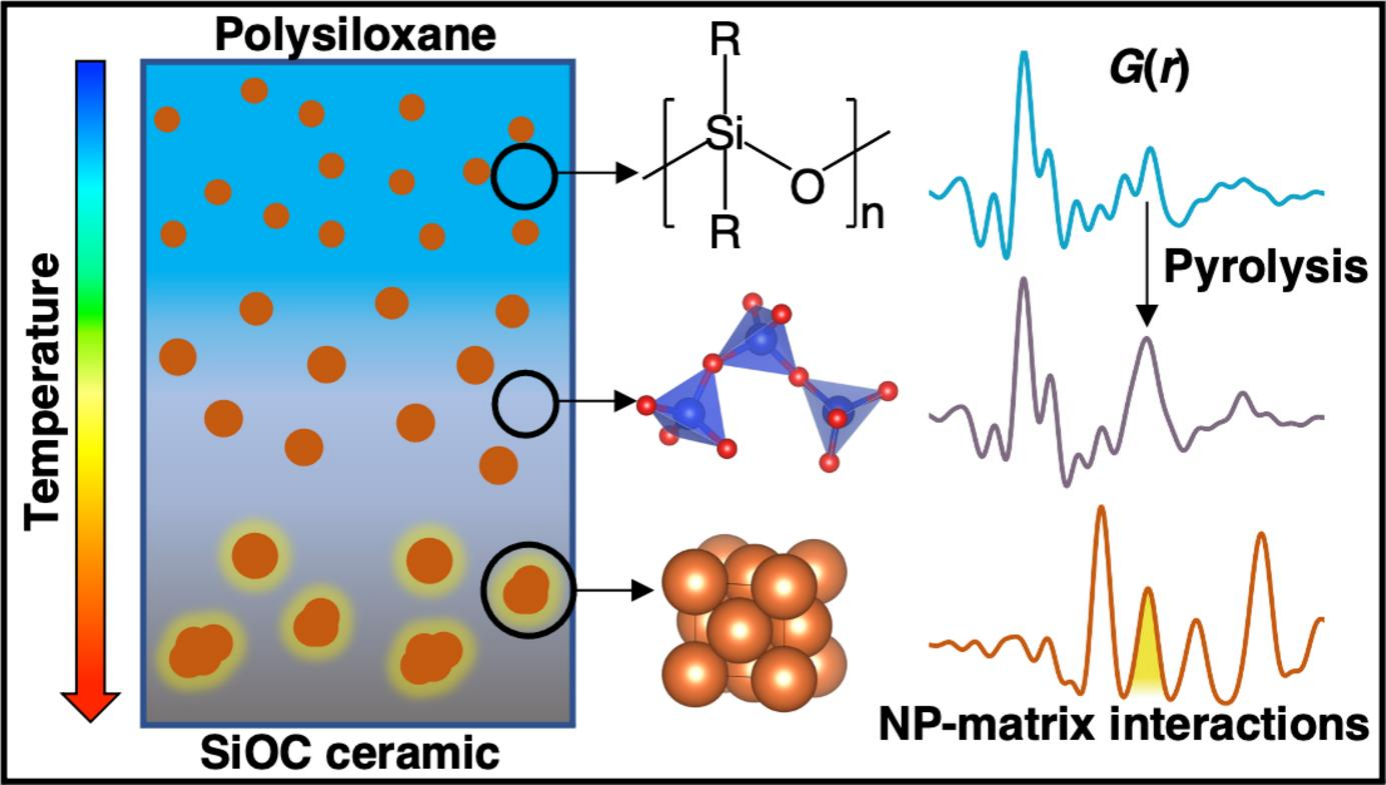
Schematic of experimental setup and PDF findings for the polysiloxane matrix before (blue) and after (gray) pyrolysis based on the transition from polymer to SiO_4_ tetrahedra, and the appearance of nanoparticle (orange) and matrix interactions (yellow) after 1000°C processing.

In this study, we present *ex situ* PDF analysis of a copper-nanoparticle-loaded commercial polysiloxane through various stages of processing up to 1000°C (Fig. 1 ▸). Additionally, for the first time we report the application of the d-PDF to monitor the interface between the copper nanofiller and a commercial polysiloxane matrix. This has huge implications for PDC nanocomposites and informs the potential of applying *in situ* PDF analysis to PCPs during processing up to high temperatures. This work opens pathways to gain further fundamental insights on the structural changes through pyrolysis at lower temperatures to amorphous and then crystalline ceramic transitions and to monitor real-time evolution about the interface between fillers and matrix, opportunities for which have been largely lacking in the literature up until now.

## 2. Materials and methods

### 2.1. Materials

The polysiloxane resin SPR-212 was purchased from Starfire Systems to serve as the PCP matrix. For the nanoparticle synthesis, copper(II) acetyl­acetonate [Cu(acac)_2_] and hexane (extra dry, 96%) were purchased from Fisher Scientific, while oleyl­amine (technical grade, 70%) and trioctylphosphine (97%) were purchased from Sigma Aldrich.

### 2.2. Copper nanoparticle synthesis

Copper nanoparticles were synthesized following an approach previously reported by Guo *et al.* (2014 ▸). Cu(acac)_2_ (0.15 mmol) was added to a three-necked flask in 7 ml of oleyl­amine. The flask was put under vacuum and purged three times before filling with Ar. Trioctylphosphine (0.462 ml) was injected into the inert reaction vessel, and the flask remained under argon for the entirety of the reaction. The contents of the three-necked flask were mixed and heated to 80°C at ramp rate of ∼4°C min^−1^. Once at 80°C, the reaction was held for 15 min before ramping (∼6°C min^−1^) to its final temperature of 200°C. The reaction was held at 200°C for 1 h before being allowed to cool naturally to room temperature. Once cooled, the solution was taken through a series of three centrifugation steps at 8000 r min^−1^ with hexane (solvent) and ethanol (antisolvent) using a Thermo Fisher Scientific Legend X1R centrifuge (7441*g* relative centrifugal field). Each time, clear solution was taken off and particles were redispersed before final storage in hexane.

### 2.3. Dispersion and SPR-212 processing

Copper nanoparticles in hexane were added dropwise to three-necked flasks containing portions of SPR-212 in four unique mass loadings (0, 2, 4 and 8 wt% added copper). During addition, the SPR-212 solutions were constantly stirred to encourage dispersion. Once the nanoparticles were added, the solutions were put under vacuum for hexane evaporation and then gently heated to 130°C to promote mixing and allow any remaining hexane to evaporate. Portions of each solution with unique copper loadings were then separated and added to alumina crucibles to undergo additional processing to 700 and 1000°C. Heat treatments were performed in a tube furnace under argon flow. The ramp rate (1°C min^−1^), final temperature dwell time (1 h) and cooling rate (5°C min^−1^) were the same for each sample. The final sample set included 12 samples (four unique copper loadings at three processing temperatures).

### 2.4. Nanoparticle characterization

Nanoparticles were characterized prior to dispersion using a mixture of transmission electron microscopy (TEM) and XRD. TEM was performed on a Field Electron and Ion (FEI) Tecnai G2-30 at 300 kV equipped with a 4k Ceta camera. XRD data were collected on a laboratory source (Cu *K*α, λ = 1.54 Å, Rigaku Miniflex 600) with Bragg–Brentano geometry. Nanoparticles dispersed in hexane were added dropwise to a glass slide, and then hexane was evaporated to leave behind a film of nanoparticles for analysis. Scans were collected in the range 2θ = 30–90° at a speed of 2° min^−1^ and step size of 2θ = 0.02°. *GSAS-II* (Toby & Von Dreele, 2013 ▸) was used to perform fits of the patterns, confirming the phase purity (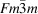 copper, ICSD-43493; Otte, 1961 ▸). Fits of the 111 peak allowed measurement of the full width at half-maximum (FWHM). The Scherrer function [equation (1)], which relates FWHM (β) to crystallite size (τ) with the Scherrer constant *K*, was used to estimate the nanoparticle size:
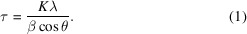


### 2.5. SPR-212 with copper nanoparticle characterization

Scanning electron microscopy (SEM) was performed for 1000°C-processed SPR-212 mixtures with all four copper loadings using a Thermo Scientific Apreo field emission gun scanning electron microscope. Images were acquired using both secondary (ETD) and backscattered electron (ABS) detectors to understand the contrast from both topography and composition. SEM energy dispersive spectroscopy (EDS) was performed using an EDAX detector and the EDAX *TEAM* software to confirm bright regions (while using the ABS detector) as Cu filler regions.

The synchrotron total X-ray scattering experiment was performed at the Advanced Photon Source 11-ID-B beamline under GUP-75644. XRD and PDF data were acquired at 1 and 180 mm sample-to-detector distances, respectively, at an X-ray wavelength of 0.2115 Å. Calibration using CeO_2_ (sample-to-detector distance) and Ni standards (instrumental peak broadening) and Rietveld refinements were performed using *GSAS-II* (Toby & Von Dreele, 2013 ▸). The measured *S*(*Q*) was transformed to the reduced-PDF following (Peterson *et al.*, 2021 ▸)
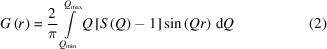
using *PDFgetX3* (Juhás *et al.*, 2013 ▸; https://www.diffpy.org/doc/pdfgui), with the maximum momentum transfer *Q*_max_ and empirical data correction term *r*_poly_ set to values of 22.8 Å^−1^ and 1.37 Å, respectively. The scattering from the Kapton sample container was subtracted during the image integration step in *GSAS-II*. For d-PDF analysis, the scale of the subtracted copper-free SiOC was adjusted manually, as there were variations in packing density across samples. For consistency, the scale factor was set to eliminate the 1.6 Å Si—O peak. Thus, any anomalous intensity in the d-PDFs should be interpreted as additional intensity relative to the Si—O peak. For renormalization and Laue diffuse scattering corrections, the sample composition was assumed to be SiOC_0.5_ for pure SPR-212 samples and pure Cu for d-PDF analysis. The sample composition was estimated by small-box fitting in *PDFgui* (Farrow *et al.*, 2007 ▸). The 4–20 Å range was fitted to face-centered cubic (f.c.c.) copper by refining the phase scale factor, lattice parameters and isotropic atomic displacement parameters. The first coordination shell of the SiOC matrix (1.0–2.7 Å) was fitted to α-SiO_2_ (ICSD-16331; d’Amour *et al.*, 1979 ▸) and 6H-SiC (ICSD-156190; Capitani *et al.*, 2007 ▸) by refining the phase scale factors, δ_2_ correlated motion factor and an isotropic strain factor. Isotropic atomic displacement parameters were fixed to 0.005 Å^2^ for silicon and 0.03 Å^2^ for oxygen and carbon. All fits were performed using crystallographic information files gathered from the International Crystal Structure Database (ICSD; https://icsd.fiz-karlsruhe.de/index.xhtml) which are referenced through­out. XRD peak identification was performed using *PDXL* (Rigaku, 2010 ▸) equipped with the International Center for Diffraction Data (ICDD) Powder Diffraction File database (Gates-Rector & Blanton, 2019 ▸).

## 3. Results and discussion

For this work, a series of SPR-212 polysiloxane samples were processed to 130, 700 and 1000°C with varying loads of copper nanoparticles to track matrix structural changes, evolution in the copper nanofiller and matrix–filler interactions. The results for processed polysiloxane with all copper loadings (0, 2, 4, 8 wt% copper) are reported below.

### 3.1. Matrix evolution

**Figure 2:**
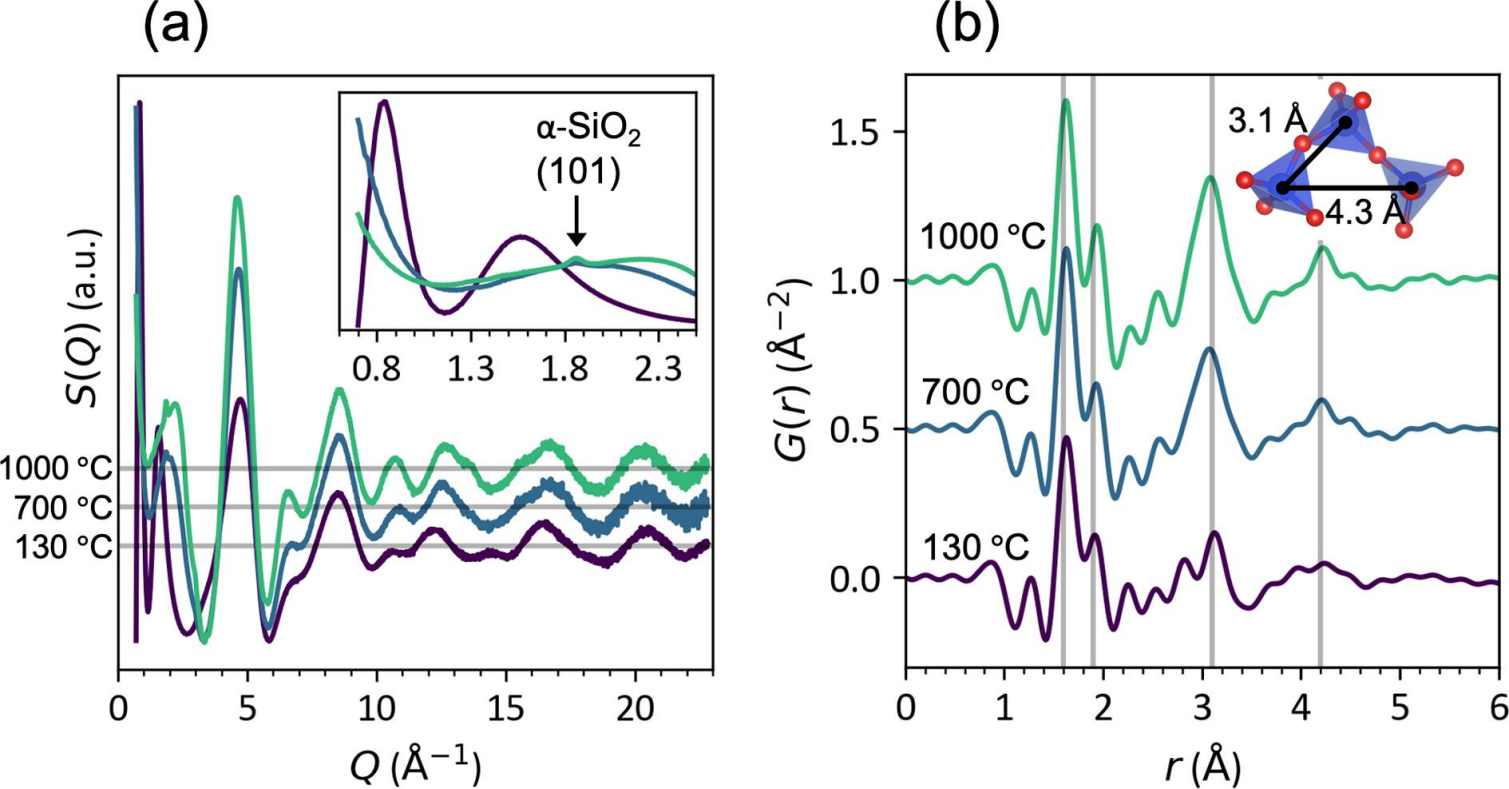
(*a*) X-ray total scattering of SPR-212 samples annealed at 130, 700 and 1000°C. The gray horizontal lines mark the *S*(*Q*) = 0 baseline. (*b*) The corresponding Fourier transform to the real-space PDF. The gray vertical lines mark the Si—O (1.6 Å), Si—C (1.9 Å) and Si—Si (3.1 and 4.2 Å) interatomic distances. The tetrahedra in the inset in (*b*) illustrate the Si—Si distances in α-SiO_2_ (ICSD-16331).

Total X-ray scattering shows the evolution of the SiOC matrix with annealing temperature. Fig. 2 ▸(*a*) shows diffuse scattering patterns of neat SPR-212 (0 wt% copper) processed at 130, 700 and 1000°C. The as-received SPR-212 and SPR-212 mixed at 130°C show identical *S*(*Q*) patterns, with no Bragg peaks from crystalline phases. Both contain a first sharp diffraction peak at 0.84 Å^−1^, characteristic of medium-range order in liquids and glasses (Terban & Billinge, 2022 ▸). After pyrolysis at 700 and 1000°C, the low-*Q* region is dominated by the start of a small-angle scattering signal indicative of nanoscale ordering (Glatter & Kratky, 1982 ▸). A small Bragg diffraction peak at 1.86 Å^−1^ from the 101 α-SiO_2_ (ICSD-16331) reflection can be seen at 1000°C, and to a lesser extent at 700°C, indicating the start of SiO_2_ nanodomain formation. The 011 α-SiO_2_ reflection is also visible in synchrotron XRD, emerging at 700°C and ripening after 1000°C processing (Fig. S1). This is typically observed at higher temperatures (1200°C) and after SiC crystallization as per the established phase separation theory for SiOC PDC processing (Kleebe *et al.*, 2001 ▸; Peña-Alonso *et al.*, 2007 ▸; Yang & Lu, 2021*a* ▸). However, β-SiC crystallization is not observed in either the 700°C- or the 1000°C-processed samples. The earlier SiO_2_ crystallization is observed because of the better signal-to-noise ratio achieved by synchrotron radiation, or possibly as a result of oxygen contamination in the processing environment (Lu & Li, 2016 ▸; Yang & Lu, 2020 ▸). Our results agree with a recent study by Lu & Chaney (2023 ▸), which also challenges phase separation theory by utilizing synchrotron XRD to capture SiO_2_ crystallization before SiC at 1200°C in a similar polysiloxane. Our results uphold that SiO_2_ crystallization happens first and put onset crystallization much earlier at around 700°C.

The diffuse scattering in the *S*(*Q*) patterns is interpreted by transforming to real-space PDFs, shown in Fig. 2 ▸(*b*), to identify frequent interatomic distances in the sample. The most intense peaks are at 1.6 and 1.9 Å from Si—O and Si—C bonds, respectively (Schiţco *et al.*, 2016 ▸). At 700 and 1000°C, there is a peak at *r* = 3.1 Å, corresponding to the Si—Si distance between corner-sharing tetrahedra in SiO_2_ and SiC. This confirms that pyrolysis of the organic PCP to the ceramic PDC can be detected by PDF analysis. It also suggests medium-range ordering in the orientation of corner-sharing SiO_4−*x*_C*_x_* tetrahedra, possibly enabled by separation into O- and C-rich domains (Saha *et al.*, 2006 ▸). This is further supported by the emergence of the peak at 4.2–4.4 Å, which suggests medium-range ordering into ring networks characteristic of amorphous SiO_2_ (Wakihara *et al.*, 2008 ▸; Rantanen *et al.*, 2019 ▸; Rino *et al.*, 1993 ▸).

### 3.2. Nanofiller ripening and dispersion

**Figure 3:**
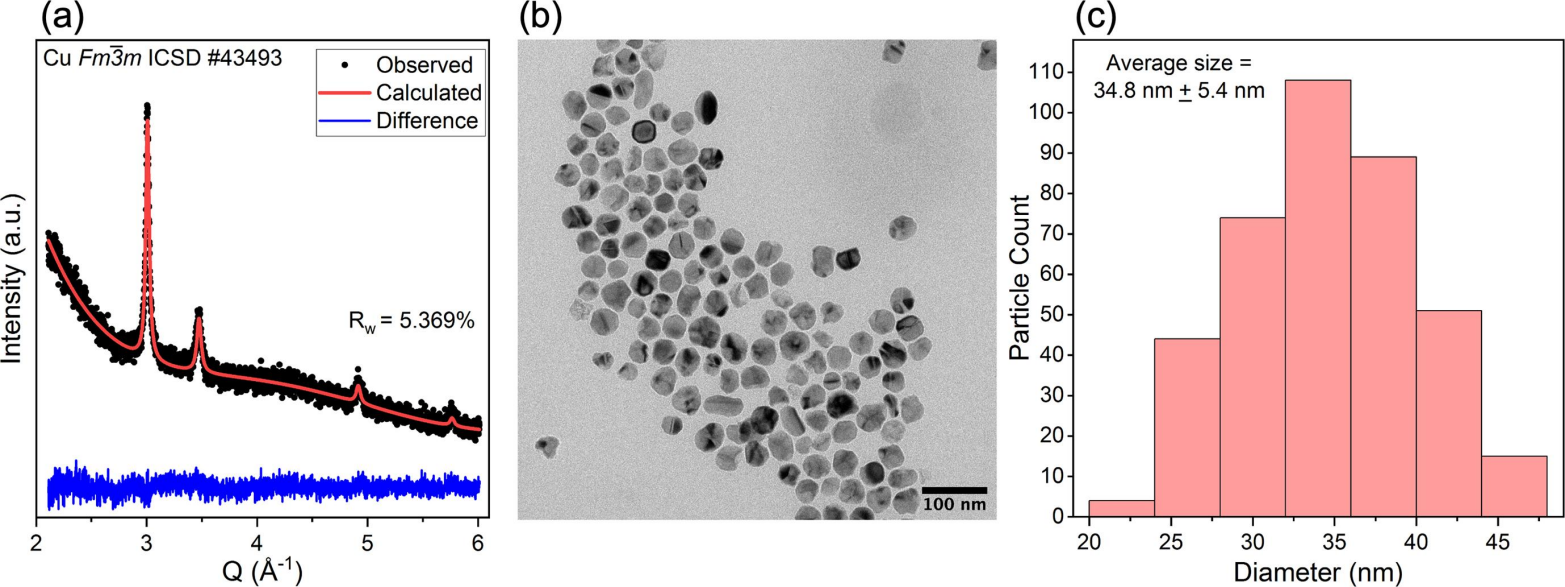
Laboratory XRD (*a*) and TEM (*b*) of as-synthesized representative copper nanoparticles with a histogram of measured sizes from TEM (*c*). The XRD data have been fitted to 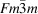 copper (ICSD-43493) for phase identification. TEM size measurements were taken from the image in (*b*) and the images in Fig. S2.

Prior to dispersion into SPR-212, copper nanoparticles were synthesized following conventional thermolysis with use of oleyl­amine as a capping ligand. Laboratory XRD and TEM were used to confirm the phase, size and morphology for nanoparticles formed following this methodology. XRD [Fig. 3 ▸(*a*)] shows four peaks indexed to the 111, 200, 220 and 311 reflections from 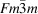 copper (ICSD-43493), confirming the phase purity of the synthesized nanoparticles. TEM [Fig. 3 ▸(*b*)] shows nanoparticles with spherical geometry and an average size of 34.8 nm [Fig. 3 ▸(*c*)].

**Figure 4:**
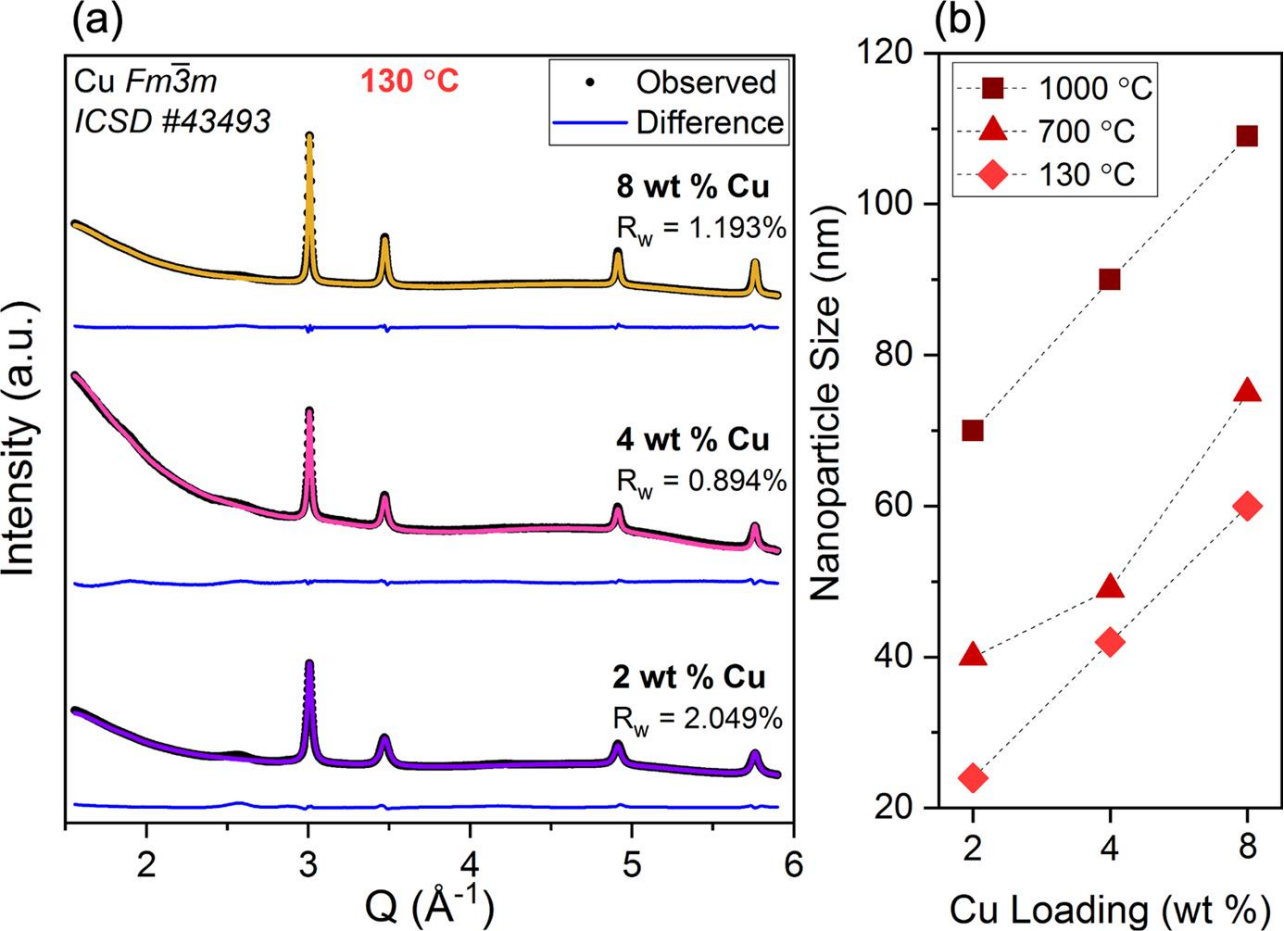
(*a*) XRD and Rietveld refinements of all Cu-containing (2, 4 and 8 wt% Cu) SPR-212 mixtures after mixing at 130°C, with (*b*) fitted nanoparticle sizes from refinements of all copper loadings processed to 130, 700 and 1000°C. The results show an increase in nanoparticle size with increased copper loading, and an increase in nanoparticle size with increasing processing temperature.

**Figure 5:**
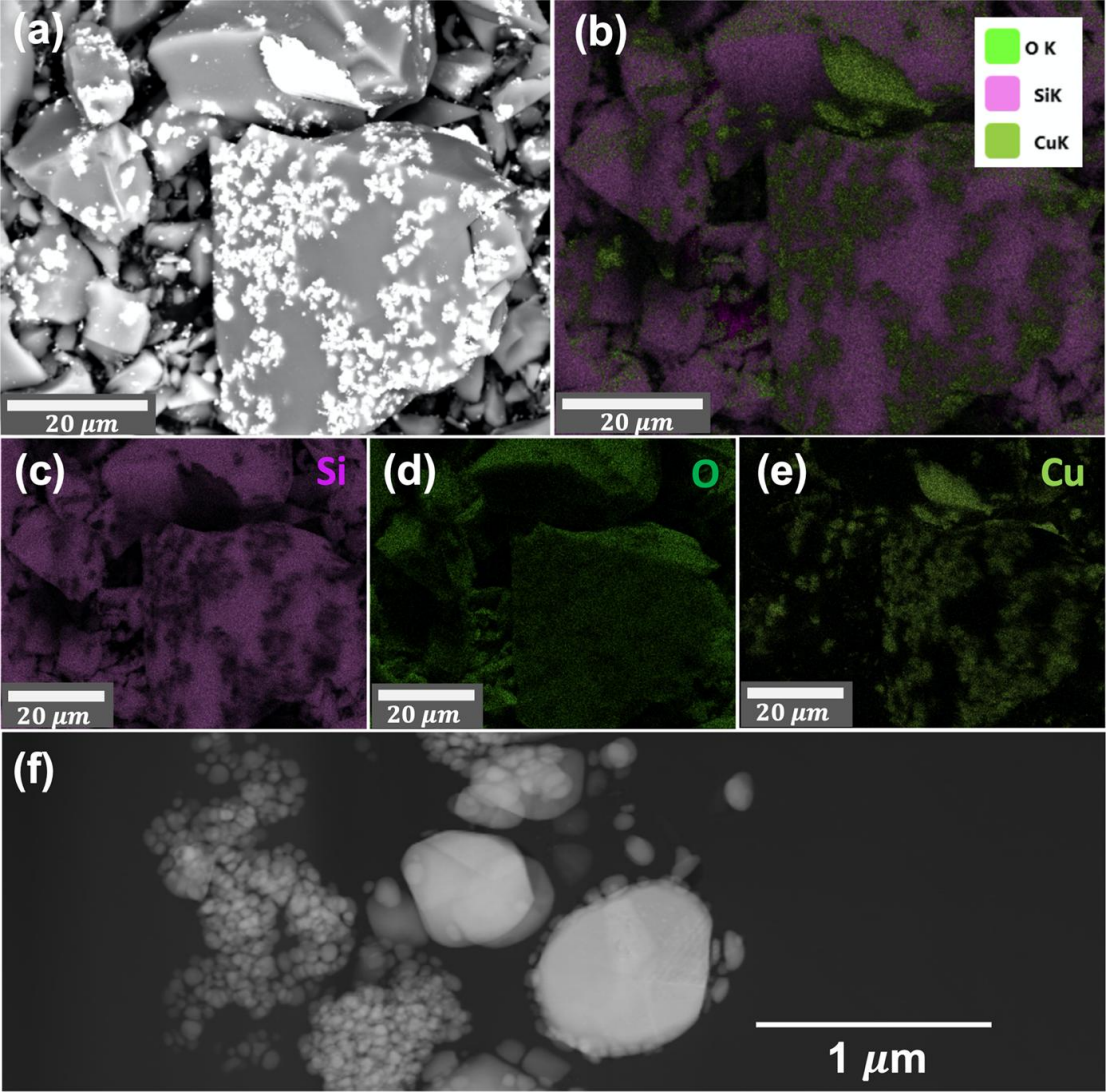
(*a*) SEM of SPR-212 with 8 wt% Cu nanoparticles after processing to 1000°C, with (*b*) the overlaid elemental mapping from EDS and (*c*, *d*, *e*) deconvolved elemental contributions from Si, O and Cu, respectively. EDS reveals the bright several-micrometre-sized regions as aggregated copper. TEM (*f*) of the same sample shows copper regions at higher magnification, confirming growth and aggregation to sizes on the order of 1 µm.

For SPR-212 with nanofillers, evolution in the crystalline nanofiller was observed with synchrotron XRD. Nanoparticle ripening with increased weight percent loading and temperature is tracked, as well as the formation of other crystalline phases at elevated temperatures. Fig. 4 ▸(*a*) compares the XRD patterns from copper-containing SPR-212 samples of all three loadings (2, 4, 8 wt% copper) after mixing at 130°C. Along with nanofiller crystalline reflections, a diffuse signal from the amorphous matrix is present in all patterns. Rietveld refinement with respect to 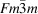 copper yields the average size estimate for the nanofiller in each copper loading and at each temperature [Fig. 4 ▸(*b*)]. The particle size was obtained from a uniaxial model with unique axis 〈111〉, because the observed 〈111〉 reflection is 1.5–2.0 times narrower than the other reflections. In f.c.c. nanoparticles, this can be attributed to 〈111〉 stacking faults and twin boundaries, which cause additional broadening for reflections outside the 〈111〉 family (Ingham *et al.*, 2011 ▸; Warren, 1990 ▸). The particle size is therefore obtained from the refined 〈111〉 axial size dimension, but this approximation probably still underestimates the particle size. The smallest fitted nanoparticle size, 23 nm for 2 wt% added copper treated at 130°C, is smaller than the average size (34.8 nm) measured by TEM for representative as-synthesized copper nanoparticles [Figs. 3 ▸(*b*) and 3 ▸(*c*)]. Regardless, the values from XRD provide qualitative trends in nanoparticle ripening throughout processing. With increased copper loading, there is a resulting increase in average particle size estimate. Thus, aggregation occurs more readily with higher mass loadings of copper. This is likely to be due to poor miscibility between the pre-ceramic polymer matrix and the nanoparticle capping ligand, oleyl­amine. This is corroborated by SEM of 1000°C-processed SPR-212 with 8 wt% copper, which reveals large bright regions surrounded by a gray matrix (Fig. S3). SEM-EDS confirms that the bright several-micrometre-sized regions are aggregated copper (Fig. 5 ▸), with growth to sizes several orders of magnitude larger than their size before pyrolysis. TEM provides a closer look [Fig. 5 ▸(*f*)], revealing some copper regions grown to sizes of ∼1 µm. This upholds the poor dispersion and apparent ripening as seen in XRD.

PDF analysis was used to estimate the weight fractions of the nanofiller and polymer matrix. The intensity of a peak in the PDF is determined by the number of atom pairs at that interatomic distance and the scattering lengths of the contributing elements. By assuming tetrahedral Si—O and Si—C coordination, we estimate the composition of the SiOC ceramic by fitting the intensity of the *r* = 1.6 Å and *r* = 1.9 Å peaks from Si—O and Si—C bonds. Likewise, this method is used to estimate the copper weight fraction. Table S1 gives SiOC composition and copper weight fraction calculated by *PDFgui* for the fits in Fig. S4. The samples appear more oxygen rich than similar SPR-212 PDCs reported by Schiţco *et al.* (2016 ▸), though values derived from the PDF are somewhat sensitive to the data reduction procedure and correlations in the fit parameters (Benmore *et al.*, 2021 ▸). Trends across samples are more reliable, and these indicate that the estimated copper weight fractions deviate from the intended mass loadings with no discernible trend. This can also be seen qualitatively from the intensity of peaks >4 Å in Fig. S4. We explain this, also, by non-uniform dispersion of nanoparticles through the matrix.

**Figure 6:**
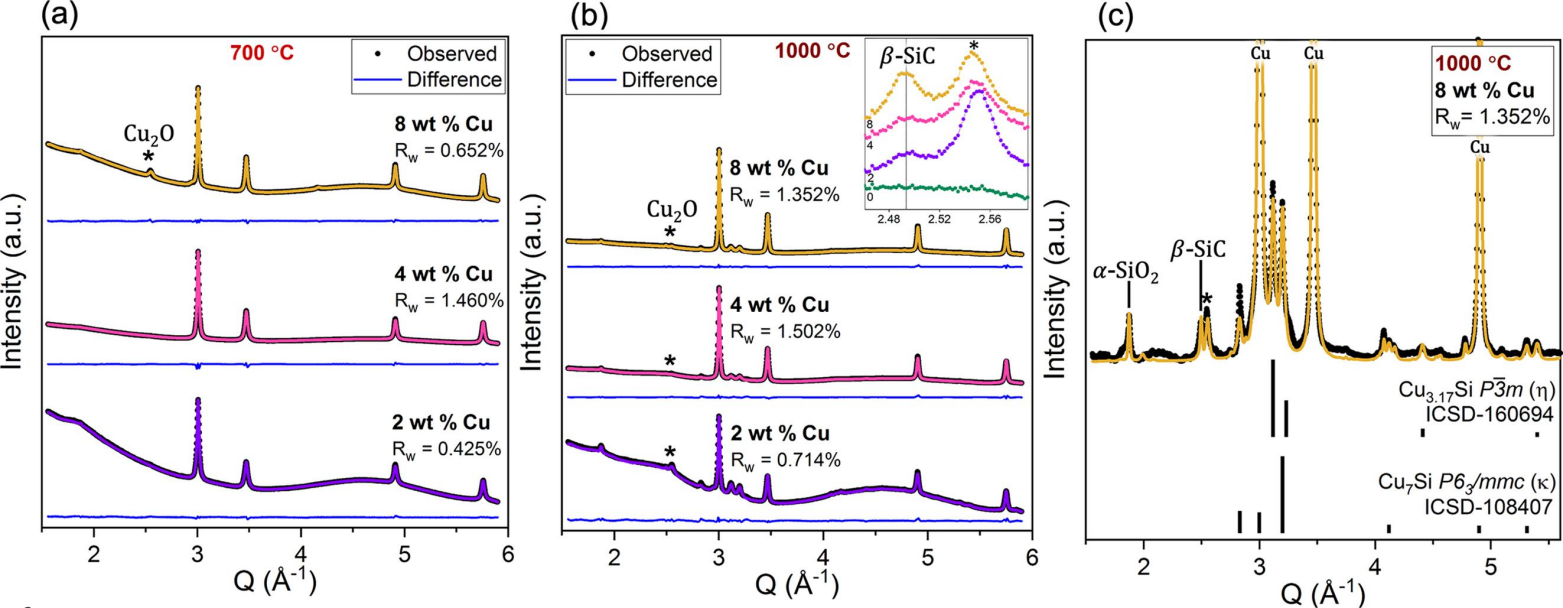
XRD and Rietveld refinement of SPR-212 with all three copper loadings after processing to (*a*) 700°C and (*b*) 1000°C, revealing an amorphous background from the matrix, f.c.c. copper peaks, occasional Cu_2_O peaks (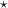) and a small 011 α -SiO_2_ (*P*3_2_21, ICSD-16331) peak at 1.87 Å^−1^. In addition to those phases, for samples processed 1000°C (*b*, *c*) two Cu–Si intermetallic species (Cu_7_Si, *P*6_3_/*mmc*, ICSD-108407 and Cu_3.17_Si, 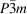, ICSD-160694) form (*c*) alongside 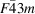 β-SiC [(*b*) inset; ICSD-24217; Braekken, 1930 ▸].

XRD was also used to monitor any new phases that appear during processing such as oxidation on a nanoparticle surface, crystallization of the matrix or intermetallic formation from matrix–particle interaction. However, this is contingent on new phase regions growing to large enough sizes to meet Bragg scattering conditions and become XRD visible. As for the neat SPR-212 samples, SiO_2_ crystallization is apparent at both 700 and 1000°C with the presence of the 011 α-SiO_2_ peak at 1.87 Å^−1^ (ICSD-16331). Copper oxidation is also visible from the emergence of the Cu_2_O (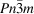, ICSD-63281; Restori & Schwarzenbach, 1986 ▸) 111 reflection at 2.55 Å^−1^ in some samples [Figs. 6 ▸(*a*) and 6 ▸(*b*)]. At 1000°C, intermetallic phases become XRD visible [Fig. 6 ▸(*b*)], with peaks indexed to the *P*6_3_/*mmc* Cu_7_Si hexagonal close-packed κ phase (ICSD-108407; Foley & Raynor, 1961 ▸) and what is likely a 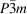 Cu_3.17_Si η phase (ICSD-160694; Wen & Spaepen, 2007 ▸) polymorph. The region where these peaks are most intense is shown in Fig. 6 ▸(*c*) with the reflection lists for the identified phases. The refined η phase structure differs slightly from the ICSD record, as fitting the most intense peak requires straining the lattice parameters by 0.7% so that the 110 and 103 reflections overlap. Regardless, the presence of intermetallic species suggests that processing at 1000°C for 1 h is sufficient for silicon to diffuse into the copper nanoparticles in quantities that influence the final microstructure. Precipitation of Cu_7_Si requires at least 10 at.% silicon, which is enough to begin melting part of the copper nanoparticles above 850°C (Rino *et al.*, 1993 ▸). The molten component provides a mechanism to form other intermetallic species, like the observed η phase which does not share a phase boundary with either Cu_7_Si or f.c.c. copper.

XRD also indicates that copper nanofillers promote the crystallization of SiC, with an increase in the 111 β-SiC reflection intensity with increasing copper loading [Fig. 6 ▸(*b*) inset]. Reflections from β-SiC at 2.50, 4.08 and 4.78 Å^−1^ [Fig. 6 ▸(*c*)] are observed in all copper-containing samples processed to 1000°C. These β-SiC peaks do not appear in the neat SPR-212 sample processed to 1000°C [Fig. S1 and Fig. 6 ▸(*b*) inset], meaning that the presence of copper and the eventual formation of intermetallic Cu–Si species must lower the critical nucleation temperature for β-SiC, which is usually ∼1200–1300°C (Rau *et al.*, 2021 ▸). This has not yet been observed for copper, but a similar phenomenon has been observed for iron additives in polysiloxane, where the formation of iron silicide promotes early crystallization of β-SiC at 1100°C and allows sites for favorable continued growth (Rau *et al.*, 2021 ▸). By using copper, Cu–Si intermetallic species are observed alongside evidence of early β-SiC crystallization at 1000°C.

### 3.3. Nanofiller–matrix interfacial study

The d-PDF method was used to identify interactions between the nanofiller and the SiOC matrix. The total scattering patterns of the copper-loaded SPR-212 samples contain Bragg peaks from the nanocrystalline filler and diffuse scattering from the SiOC matrix. On subtracting the diffuse scattering of the SiOC sample without copper, the remaining signal is from the copper nanofiller and its interactions with the SiOC matrix. The d-PDFs at 130 and 700°C [Fig. S4(*a*) and S4(*b*)] fit well to pure f.c.c. copper, suggesting that the copper particles are functioning as passive fillers at lower processing temperatures.

**Figure 7:**
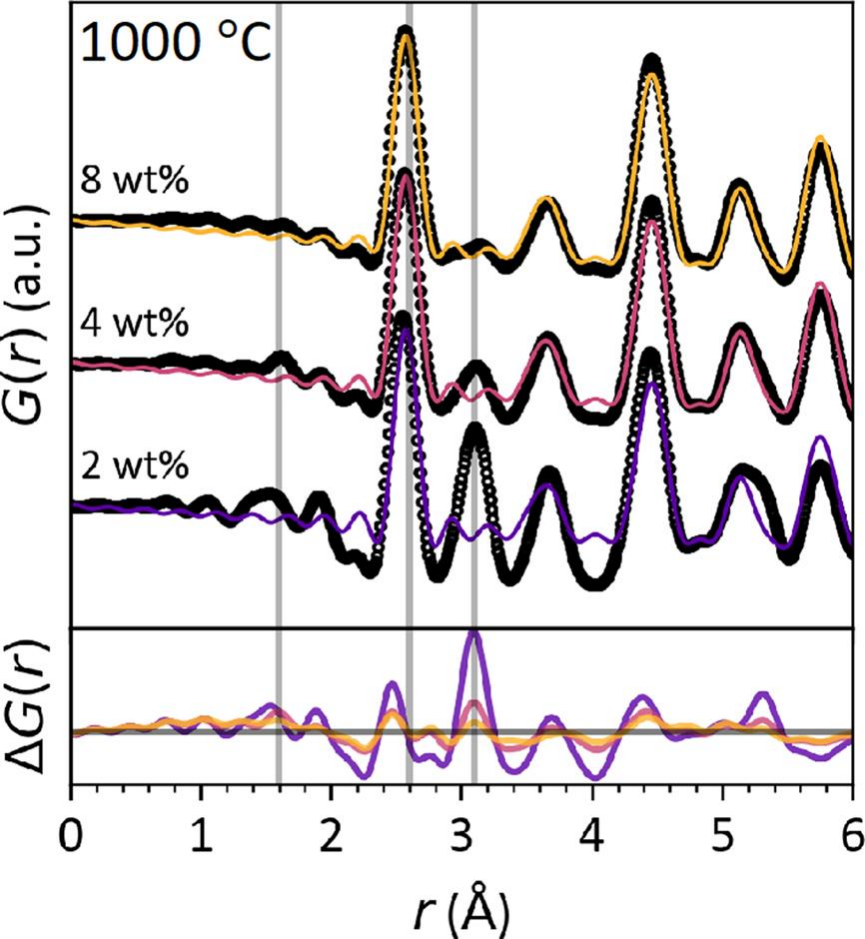
Differential PDF of Cu-SPR-212 samples annealed at 1000°C. The vertical lines mark the Si—O peak (1.6 Å) eliminated by subtracting the SiOC matrix scattering, the typical M—M (M = Cu, Si) distance (2.6 Å) in Cu-rich metals and the typical M—M distance (3.1 Å) in Cu/Si oxides.

In all samples processed at 1000°C (Fig. 7 ▸) and the 700°C sample with 8 wt% copper, the d-PDF shows an anomalous peak at 3.1 Å. This interatomic distance is not present in the identified intermetallic species and not likely to appear in any metallic phase that maintains a ∼2.6 Å bond distance. Rather, this corresponds to the typical metal–metal interatomic distance in silicon and copper oxides. These are the same samples with detectable Cu_2_O Bragg peaks [see Figs. 6 ▸(*a*) and 6 ▸(*b*)], which suggests that this anomalous d-PDF peak is from oxidation of the copper nanoparticles. However, the crystalline Cu_2_O content is not correlated to the anomalous intensity in the d-PDF. Rietveld refinements estimate that the weight fraction of oxidized copper is similar for the 700°C, 8 wt% and 1000°C, 2 wt% samples, but the intensity of the 3.1 Å d-PDF peak is significantly larger in the latter (see Fig. S5). The relationship is true for the 1000°C, 8 wt% and 4 wt% samples. If this peak is from copper oxide, it must be amorphous or confined to nano-sized domains, probably at the interface between the nanoparticles and the SiOC matrix. A surface effect is consistent with the higher relative intensity in samples with smaller average nanoparticle size. Alternatively, this trend may point to a change in the bulk SiOC matrix. This is possible because copper has a relatively high diffusivity in SiO_2_ and oxygen-rich SiOC (Ming *et al.*, 2019 ▸; Thermadam *et al.*, 2010 ▸; Dallaporta *et al.*, 1990 ▸; Koh *et al.*, 2003 ▸). The appearance of the 4.2 Å peak in Fig. 2 ▸ suggests that there are tetrahedral SiO_4_ ring networks that could accommodate copper interstitials, which could produce the observed d-PDF peak by locally ordering to maintain the 3.1 Å metal–metal interatomic distances typical of the bulk oxides. A similar feature was observed in d-PDF analysis of caesium-intercalated zeolite SiO_2_ (Petkov *et al.*, 2002 ▸). Distinguishing between amorphous Cu_2_O and Cu interstitials may be possible from analyzing the Cu—O bond distance, but owing to differences in packing density across samples, this peak had to be eliminated to normalize the SiOC matrix subtraction. To avoid this loss of information in future d-PDF studies, the packing density of each sample should be carefully measured.

Regardless of the source of the anomalous d-PDF peak, the high diffusivity of copper in the SiOC matrix may also contribute to nanoparticle agglomeration. This fast diffusion has previously been intentionally utilized to form copper nanostructures by thermally annealing copper-ion-implanted SiO_2_ and SiOC (Ming *et al.*, 2019 ▸; Pan *et al.*, 2007 ▸; Shirokoff *et al.*, 2010 ▸). For PDC applications, copper diffusion and agglomeration may be mitigated by optimizing the C:O ratio in the SiOC matrix, as carbon-rich SiOC films have been shown to suppress copper diffusion in electronics applications (Koh *et al.*, 2003 ▸; Thermadam *et al.*, 2010 ▸).

## 4. Conclusions

Total X-ray scattering is used to track neat matrix evolution and differentiate between interfacial interaction, nanofiller and matrix changes in nanoparticle-containing SPR-212. For the neat matrix, Bragg peaks reveal the start of SiO_2_ crystallization at low temperatures and before the start of SiC crystallization owing to the high resolution achievable with synchrotron radiation. With PDF analysis, we have probed local structure at much earlier temperatures than is possible with XRD through the presence of Si—O and Si—C bonds in the pre-ceramic polymer backbone. After annealing to 700°C, we begin to see a ripening of bond distances that match up with Si—Si distances in SiC and SiO_2_. We attribute this to the formation of corner-sharing Si–O–C tetrahedra, indicating that pyrolysis to amorphous ceramic has occurred. When including copper nanofiller into SPR-212, the nanoparticles readily aggregate upon mixing, probably because of unfavorable thermodynamics of mixing between the polymer matrix and capping ligand, and ripen with increasing processing temperature. After 1000°C processing, we see the formation of two unique Cu–Si species: a Cu-rich species (Cu_7_Si) and a relatively Si-rich species (Cu_3.17_Si). Along with Cu–Si intermetallic species, SiC also begins to crystallize after 1000°C processing in all copper-containing samples. This is not observed in the neat matrix and occurs at much lower temperatures than expected for SiC nucleation. The formation of Cu–Si intermetallic species must lower the critical nucleation temperature for SiC formation. By subtracting the scattering signal of the neat matrix at each temperature from that of copper-dispersed SPR-212, we produce a d-PDF signal with contributions only from copper nanofiller and its interactions with the polysiloxane matrix. The results of this analysis point to copper acting as more than a passive filler when processed at 1000°C, with either the formation of a copper oxide interphase or copper diffusion into oxygen-rich regions of the matrix. Measurements of mechanical and electronic properties may clarify which process occurs and how the nanofiller influences the functional properties of the material.

With this work, we have demonstrated that PDF analysis can probe the local structure of a PCP before and after pyrolysis, with prevalent backbone bonds appearing in the polymeric state and evidence of SiOC tetrahedra appearing post-pyrolysis. We have also used X-ray scattering and PDF analysis to track changes in the PDC-dispersed nanofiller, including nanofiller ripening through processing and dispersion. The impact of applying *ex situ* synchrotron X-ray scattering and PDF analysis throughout different stages of processing informs future work that could extend to *in situ* structural studies. Perhaps most impactful, however, is our application of the d-PDF to isolate interfacial reactions that occur between the nanofiller and PDC matrix and the mechanism by which these interfacial reactions occur. Herein we present *ex situ* characterization of one specific PDC and nanofiller system. However, the impact of this study extends far beyond commercial polysiloxane with copper filler. The modulation of the PCP backbone and nanofiller chemistry, size and morphology shows promise to profoundly influence the final structure and properties. In this work, we have provided a characterization pathway that will inform these convoluted structural transitions for the vast variety of highly tailorable systems achievable in the PDC community.

## Supplementary Material

Crystal structure: contains datablock(s) Cu-SPR212_Rietveld_IUCr_SiC_SiO2_publ, Cu-SPR212_Rietveld_IUCr_SiC_SiO2_overall, Cu-SPR212_Rietveld_IUCr_SiC_SiO2_phase_2, Cu-SPR212_Rietveld_IUCr_SiC_SiO2_phase_3, Cu-SPR212_Rietveld_IUCr_SiC_SiO2_phase_1, Cu-SPR212_Rietveld_IUCr_SiC_SiO2_phase_0, Cu-SPR212_Rietveld_IUCr_SiC_SiO2_phase_4, Cu-SPR212_Rietveld_IUCr_SiC_SiO2_phase_5, Cu-SPR212_Rietveld_IUCr_SiC_SiO2_pwd_0, Cu-SPR212_Rietveld_IUCr_SiC_SiO2_pwd_1, Cu-SPR212_Rietveld_IUCr_SiC_SiO2_pwd_2, Cu-SPR212_Rietveld_IUCr_SiC_SiO2_pwd_3, Cu-SPR212_Rietveld_IUCr_SiC_SiO2_pwd_4, Cu-SPR212_Rietveld_IUCr_SiC_SiO2_pwd_5, Cu-SPR212_Rietveld_IUCr_SiC_SiO2_pwd_6, Cu-SPR212_Rietveld_IUCr_SiC_SiO2_pwd_7, Cu-SPR212_Rietveld_IUCr_SiC_SiO2_pwd_8, Cu-SPR212_Rietveld_IUCr_SiC_SiO2_pwd_9, Cu-SPR212_Rietveld_IUCr_SiC_SiO2_pwd_10, Cu-SPR212_Rietveld_IUCr_SiC_SiO2_pwd_11. DOI: 10.1107/S1600576724003133/iu5053sup1.cif

Supporting figures and table. DOI: 10.1107/S1600576724003133/iu5053sup2.pdf
