# Supplementary material for: Tracking copper nanofiller evolution in polysiloxane during processing into SiOC ceramic
Source: J Appl Crystallogr. 2024 Jun 18;57(Pt 4):945–54. doi: 10.1107/S1600576724003133 (PMC11299600; doi:10.1107/S1600576724003133)
Supplement: Supplementary file 2 [file j-57-00945-sup2.pdf]

JOURNAL OF
APPLIED
CRYSTALLOGRAPHY

Volume 57 (2024)

Supporting information for article:

Tracking copper nanofiller evolution in polysiloxane during processing into SiOC ceramic

Patricia A. Loughney, Paul Cuillier, Timothy L. Pruyn and Vicky Doan-Nguyen

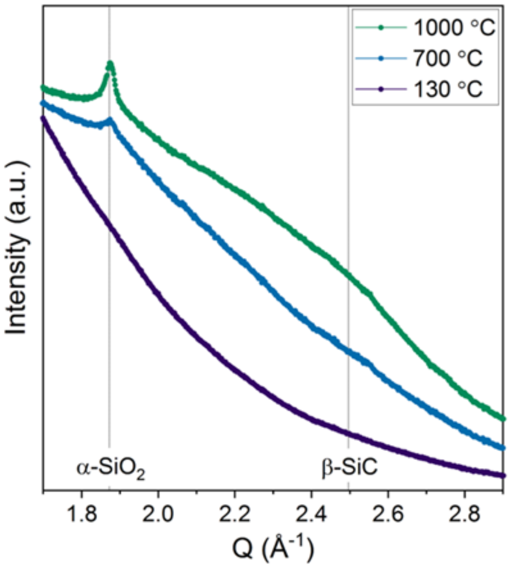

Figure S1 Synchrotron XRD of neat SPR-212 (0 wt % Cu) after processing to 130 °C, 700 °C, and 1000 °C. After processing to 130 °C, no Bragg peaks are apparent over the given Q-range. By 700 °C, the 011 α-SiO_2 (ICSD-16331) appears and ripens after 1000 °C processing indicating α-SiO_2 crystallization. The β-SiC 220 parent reflection Q-value (ICSD-24217) is also monitored, though does not appear despite 1000 °C processing and SiO_2 crystallization.

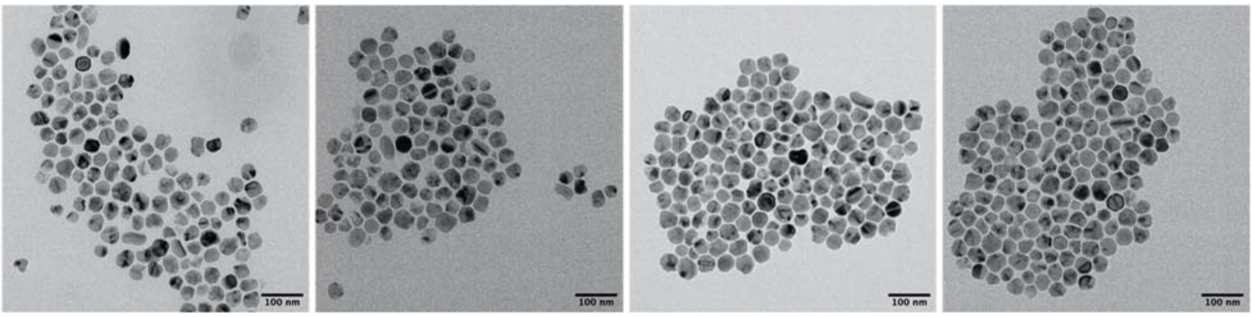

Figure S2 Additional TEM of copper nanoparticles post-synthesis. A histogram of nanoparticle size measured from these images is reported in Figure 2c.

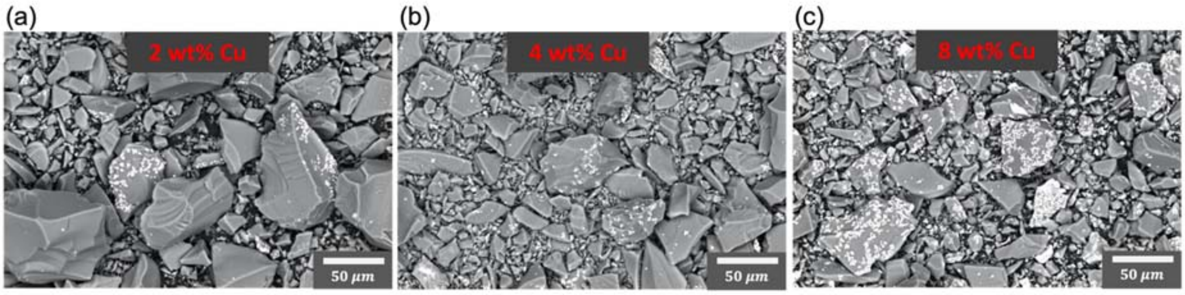

Figure S3 Low magnification SEM of all Cu-containing (2, 4, 8 weight % Cu) SPR-212 samples after processing to 1000 °C.

Table S1 SiOC matrix composition and copper weight percent for each sample, estimated by the PDF fits in Figure S4.

These values rely on the assumption Si is tetrahedrally coordinated, which is not true at 130 °C before pyrolysis. This is primarily to illustrate the variation in estimated Cu loading is not consistent with the known mass loading.

T (°C)	Added Cu wt%	SiOC Composition	Est. Cu wt%
130	0	$SiO_{1.29}C_{0.36}$	
	2	$SiO_{1.29}C_{0.35}$	8.0
	4	$SiO_{1.47}C_{0.27}$	3.2
	8	$SiO_{1.58}C_{0.21}$	7.7
700	0	$SiO_{1.39}C_{0.31}$	
	2	$SiO_{1.57}C_{0.22}$	2.8
	4	$SiO_{1.60}C_{0.20}$	10.3
	8	$SiO_{1.74}C_{0.13}$	4.4
1000	0	$SiO_{1.35}C_{0.32}$	
	2	$SiO_{1.48}C_{0.26}$	2.1
	4	$SiO_{1.52}C_{0.24}$	9.8
	8	$SiO_{1.49}C_{0.26}$	11.5

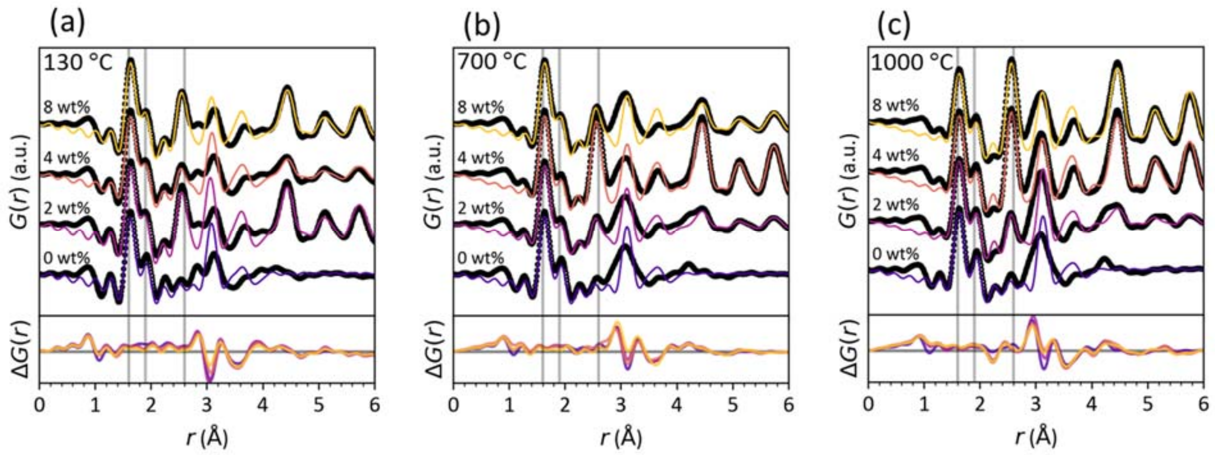

Figure S4 PDF of copper-loaded SPR-212 processed at (a) 130 °C, (b) 700 °C, and (c) 1000 °C. The vertical gray lines mark the peaks from Si-O, Si-C, and Cu-Cu interatomic distances, fit to SiO_2, SiC, and Cu, respectively.

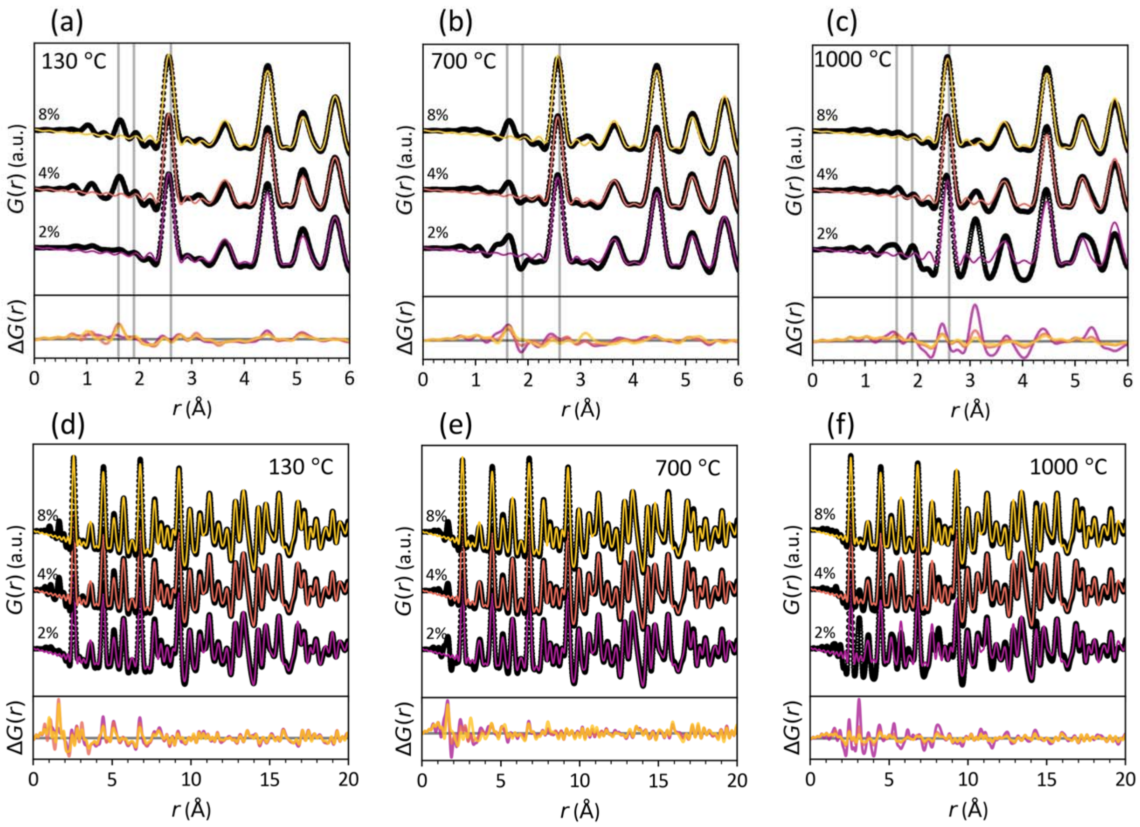

Figure S5 d-PDF at (a, d) 130 °C, (b, e) 700 °C, and (c, f) 1000 °C. ΔG(r) is the difference between the d-PDF and the fit to FCC copper, with all panels on the same scale. The marked interatomic distances (a-c) correspond to the Si-O/Cu-O distance used to normalize the background subtraction for obtaining the d-PDFs, the Cu-Cu distance in FCC Cu, and the anomalous peak at 3.1 Å. The same d-PDF fits at (d) 130 °C, (e) 700 °C, and (f) 1000 °C shown out to 20 Å to emphasize that the deviation from pure copper is primarily in the short-range order region.
